# An Expert Guide to Anatomy-Based Filler Injection for the Temple: Techniques and Clinical Insights

**DOI:** 10.3390/life15020266

**Published:** 2025-02-10

**Authors:** Gi-Woong Hong, Jovian Wan, Wonseok Choi, Kyu-Ho Yi

**Affiliations:** 1 Samskin Plastic Surgery Clinic, Seoul 06577, Republic of Korea; 2 Medical Research Inc., Wonju, Republic of Korea; 3 V Plastic Surgery, Daegu, Republic of Korea; 4 Division in Anatomy and Developmental Biology, Department of Oral Biology, Human Identification Research Institute, BK21 FOUR Project, Yonsei University College of Dentistry, 50-1 Yonsei-ro, Seodaemun-gu, Seoul 03722, Republic of Korea; 5 You & I Clinic (Mokdong), Seoul 06001, Republic of Korea

**Keywords:** temporal region, dermal fillers, anatomy, facial nerve, injections, subcutaneous

## Abstract

Temporal hollowing is a common aesthetic concern addressed with filler injections using an anatomy-based approach. Understanding the complex anatomy of the temporal region, including the superficial temporal artery, temporal branch of the facial nerve, and sentinel vein, is essential for safe and effective treatment. Injection planes—subfascial, within the superficial temporal fat pad, or submuscular—are selected based on individual anatomy and desired outcomes. Techniques like retrograde horizontal fanning with a cannula optimize filler distribution, enhance contour, and minimize complications. Recognizing zones of caution helps prevent vascular and nerve injuries. This approach allows clinicians to achieve natural, youthful fullness in the temples, improving overall facial aesthetics and patient satisfaction.

## 1. Introduction

Temporal hollowing is a common aesthetic concern that can result from aging, weight loss, or genetic predisposition, leading to a sunken appearance in the temples. This condition significantly impacts facial harmony, often making the face look gaunt or older. The temporal region’s anatomy, defined by its complex layers of fat, fascia, muscles, and vascular structures, presents unique challenges for filler injections. Proper identification of the injection planes—subfascial, within the superficial temporal fat pad, or submuscular—is crucial to achieving safe and effective outcomes. Understanding the intricate anatomy of the temporal area, including the location of vital structures like the superficial temporal artery, temporal branch of the facial nerve, and sentinel vein, helps minimize risks and enhance treatment precision [1,2,3,4,5].

An anatomy-based approach to filler injection in the temporal region prioritizes safety while maximizing aesthetic results. Techniques such as retrograde horizontal fanning with a cannula are commonly employed to deliver filler precisely between anatomical layers, providing volume restoration and smooth contours. Additionally, recognizing zones of caution, such as the area surrounding the sentinel vein and temporal nerve branches, is critical to avoiding complications. This comprehensive understanding of the temporal anatomy enables clinicians to tailor injection techniques to the unique needs of each patient, restoring youthful fullness and enhancing overall facial aesthetics [6].

This paper serves as an expert guide that synthesizes anatomical knowledge and clinical expertise for safe and effective filler injection techniques in the temple area. Unlike a systematic review or an empirical anatomical study, our approach integrates established anatomical insights with hands-on clinical strategies developed from extensive practical experience. By bridging anatomical understanding with applied injection methods, we aim to offer clinicians a comprehensive framework that not only enhances aesthetic outcomes but also prioritizes patient safety. This expert opinion piece is grounded in anatomical studies and clinical observations to provide actionable guidance for practitioners addressing temporal hollowing.

## 2. Pre-Procedural Considerations

**Figure 1:**
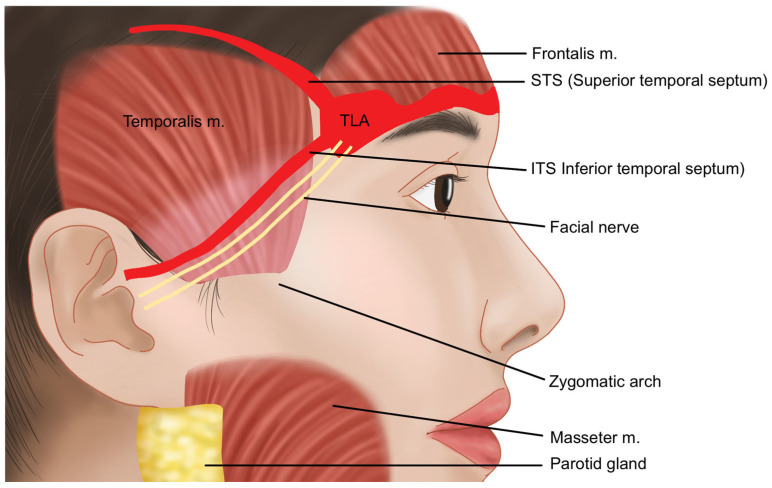
Boundary of the temporal region.

**Figure 2:**
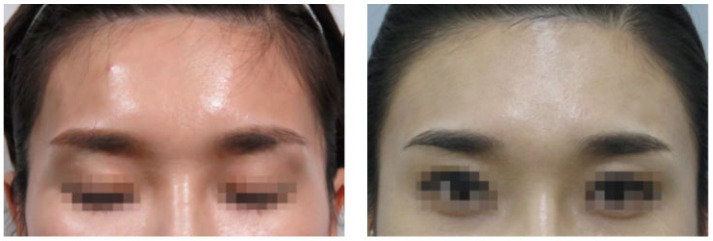
Before and after treatment of the temple filler injection.

The temple area refers to the hollow region located below the superior temporal line (STL) or superior temporal septum (STS), where the temporalis muscle originates (Figure 1) [7,8,9]. In individuals of Korean descent, the temple area often lacks natural volume, which can become more pronounced with age. The prominent zygomatic bones and zygomatic arch can exacerbate the sunken appearance of the temples, leading to a more gaunt and aged look [10]. By restoring volume to the sunken temple area with filler injections, the transition from the lateral forehead above to the zygomatic arch below can be softened, resulting in a smoother lateral facial contour. This approach helps to alleviate the harsh and aged appearance associated with hollow temples, contributing to a more youthful and harmonious facial profile (Figure 2) [11,12,13].

## 3. Anatomical Considerations

Successful volumization of the temple area requires a thorough understanding of the anatomical layers involved. The temporal fossa, which forms the base of the temple area, is a depression on the side of the skull corresponding to the temporal bone. The temporalis muscle, which lies atop the temporal fossa, is composed of two layers: the superficial layer and the deep layer, with the temporalis tendon situated between them. Within the muscle, the deep temporal artery and vein traverse [14,15,16].

Covering the temporalis muscle are two fascial layers. The upper fascial layer is known as the superficial temporalis fascia (STF), also referred to as the temporoparietal fascia (TPF). This fascia envelops the superficial temporal artery and vein and continues superiorly with the frontalis muscle (forehead), laterally with the galea aponeurotica (scalp), and inferiorly with the superficial musculoaponeurotic system (SMAS) of the midface. The lower fascial layer, known as the deep temporal fascia (DTF), also called the temporalis fascia or temporalis muscle fascia, originates from the superior temporal septum (STS). As it descends, the DTF splits into two layers—superficial and deep—which rejoin approximately 1 cm above the zygomatic arch to envelop the arch both anteriorly and posteriorly, though individual variations can occur [12,17,18,19,20].

Within the spaces created by these layered structures, several important anatomical features can be found. The deep temporal fat pad (DTFP), which is an extension of the buccal fat pad, is located between the temporalis muscle and the DTF. The superficial temporal fat pad (STFP), or simply temporal fat pad, resides between the superficial and deep layers of the DTF, typically extending 3 to 4 cm above the zygomatic arch. The compartmentalized space between the STF and DTF is divided by the inferior temporal septum (ITS) into two distinct areas: the upper and lower temporal compartments (Table 1) [21].

**Figure 3:**
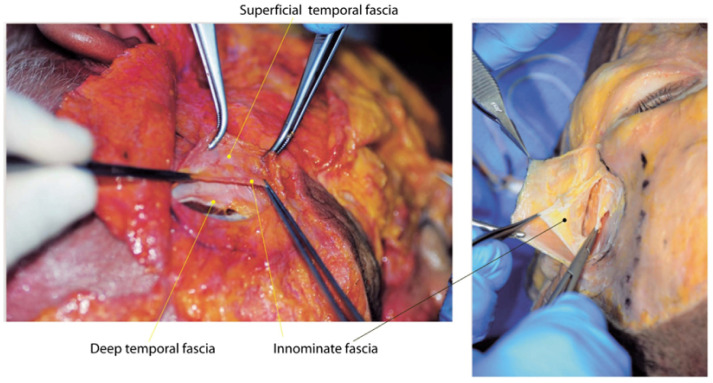
Innominate fascia of the temporal region.

Recent studies suggest the presence of an additional, consistently separated fascia within the compartments of the temple area. This unnamed fascia, referred to as the “innominate fascia” due to the lack of an official designation, can be observed as a distinct layer in cadaveric dissections of the temple region, as shown in Figure 3. Some anatomists propose that this fascia extends anatomically to connect with similar structures in the forehead and lateral cheek areas [22].

Above the fascial layers, as in other regions of the face, lie the subcutaneous fat layer and skin. When performing procedures in the temple area, particular attention must be paid to the vessels and nerves that traverse this region. These include the anterior branch of the superficial temporal artery (STA), the zygomatico-orbital artery, the middle temporal vein, the sentinel vein, the temporal branch of the facial nerve (a motor nerve), and the zygomatico-temporal nerve (a sensory nerve) [23,24]. The relationship between these anatomical structures and the two compartments of the temple region warrants careful consideration during interventions [17].

The upper temporal compartment is an enclosed structure, bordered by the STS (superior temporal septum) and ITS (inferior temporal septum), which are extensions of the temporal ligament adhesion—a firm structure that continues from the supraorbital ligamentous adhesion. This compartment is composed solely of soft tissue and lacks significant blood vessels or nerves, making it a safe area for procedures such as anchoring during thread lifting [25,26,27,28].

**Figure 4:**
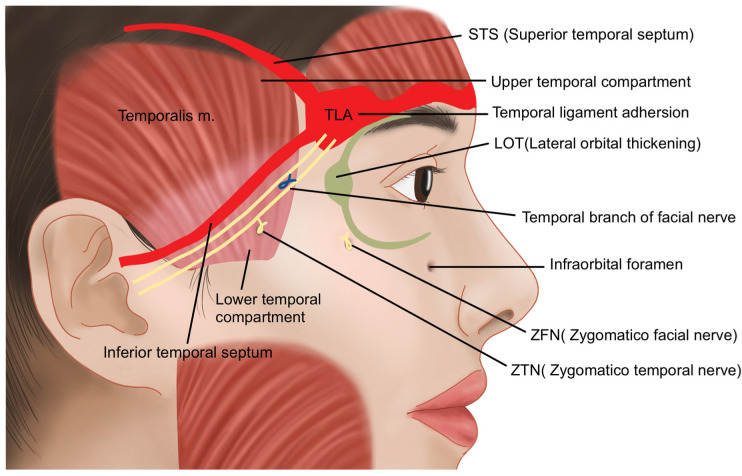
Comparison of the upper and lower compartments of the temporal region.

In contrast, the lower temporal compartment is a triangular-shaped area formed below the ITS. Unlike the upper temporal compartment, which contains minimal fat tissue, the lower temporal compartment has a higher concentration of fat, especially as it extends closer to the zygomatic arch. While the upper boundary is sealed by the ITS, the lower boundary adjacent to the zygomatic arch is not enclosed, allowing the passage of important blood vessels and nerves. These include the temporal branch of the facial nerve, the medial and lateral branches of the zygomatico-temporal nerve (ZTN), and the sentinel vein. Notably, the temporal branch of the facial nerve has been traditionally understood to run almost parallel to the boundary of the ITS; however, cadaver studies reveal that there is considerable individual variation in its course (Figure 4) [29].

**Figure 5:**
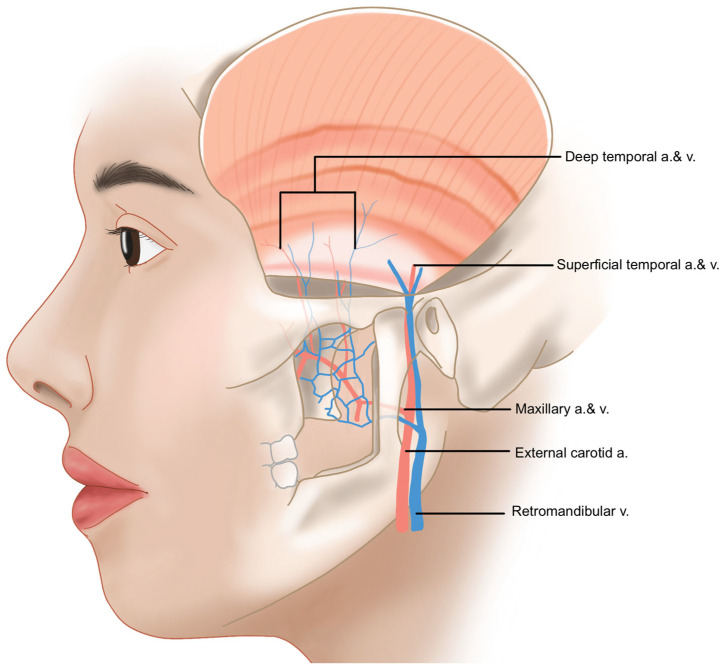
Origin of the superficial and deep temporal artery and vein.

The blood supply to the temple area is primarily provided by the superficial temporal artery (STA) and the deep temporal artery (DTA). The STA branches directly from the external carotid artery (ECA) and is responsible for the vascularization of the temple, forehead, and scalp. In contrast, the DTA originates from the maxillary artery, a branch of the ECA, and supplies blood to the temporalis muscle within the temple area (Figure 5) [30].

**Figure 6:**
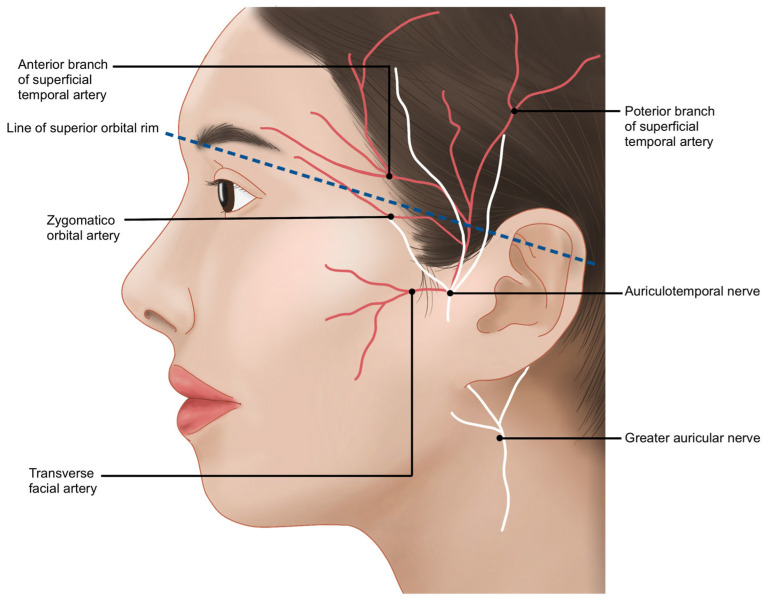
Pathway of the superficial temporal artery.

The STA ascends almost vertically along the preauricular crease, located anterior to the tragus of the ear. Near the level of the superior orbital rim, it bifurcates into anterior and posterior branches. The anterior branch travels at an average angle of approximately 60° in a superomedial direction, enclosed by the superficial temporalis fascia (STF). It enters the lateral margin of the frontalis muscle, about 1.5 to 2 cm above the lateral end of the eyebrow. Medially, the STA remains within the frontalis muscle until it crosses the vertical line drawn from the lateral canthus, where it gradually becomes more superficial and sends branches into the subcutaneous tissue (Figure 6) [30].

**Figure 7:**
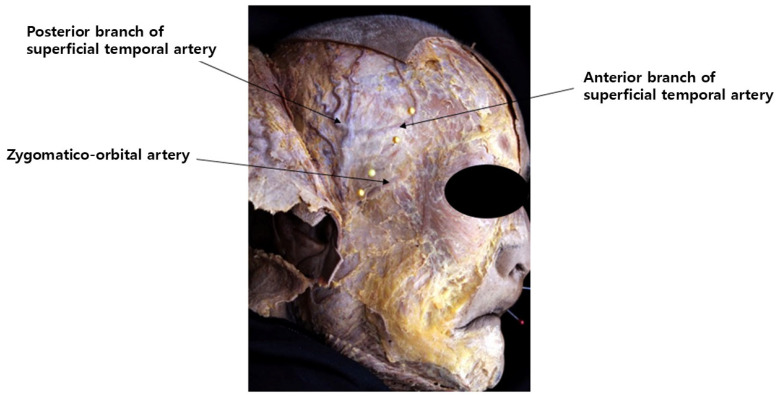
Location of the zygomatico-orbital artery.

**Figure 8:**
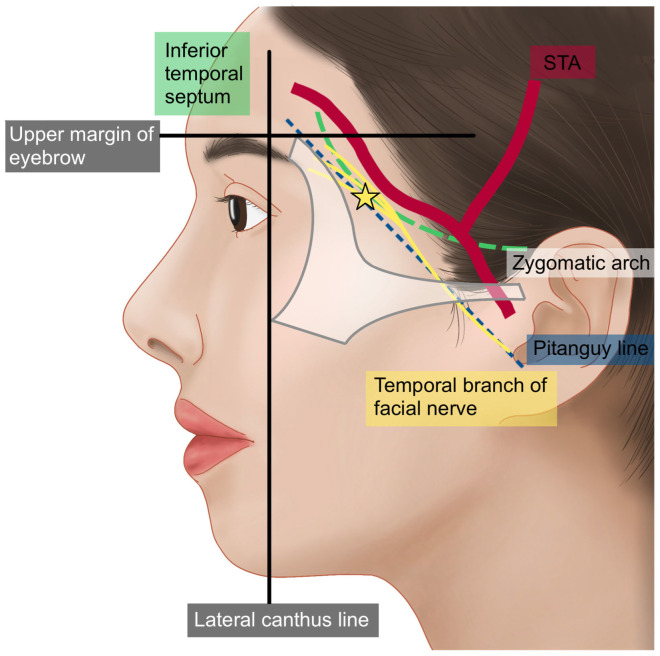
Pathway of the temporal branch of the facial nerve.

Another significant artery running from the outer temple area towards the forehead is the zygomatico-orbital artery, often simply referred to as the orbital artery. This artery branches from the main trunk of the superficial temporal artery (STA) before it divides into its anterior and posterior branches. It is reported to be present in approximately 80% of Koreans [29]. Typically, the zygomatico-orbital artery emerges near the upper margin of the zygomatic arch, traveling obliquely towards the lateral canthus and then gradually ascending to pass above the lateral end of the eyebrow (Figure 7) [30]. Regarding the course of motor nerves in the temple area, the temporal branch of the facial nerve, as previously mentioned, is generally understood to run parallel to the inferior temporal septum (ITS). However, in practice, multiple irregular branches are present, mainly located within a triangular area formed by three points: the junction of the ear lobule and cheek, the lateral end of the eyebrow, and the hairline at the boundary between the forehead and temple. Most branches are situated inferomedially to the anterior branch of the STA [14]. Additionally, the course of these nerves can be approximated using Pitanguy’s line, a virtual line drawn between a point 0.5 cm below the tragus and a point 1.5 cm lateral to the lateral margin of the eyebrow (Figure 8) [22].

In clinical practice, it is crucial to understand the precise point where the zygomatico-orbital artery crosses the zygomatic arch. Anatomically, this crossing typically occurs within a region defined by two key points: approximately 1.8 cm from the junction of the ear and cheek at the posterior boundary, and about 2 cm from the lateral orbital rim margin at the anterior boundary. This artery traverses a space approximately 8 mm wide between these two points. After crossing the zygomatic arch, it ascends to enter the frontalis muscle approximately 2 cm above the lateral end of the eyebrow. Clinically, this nerve branch can be visualized as passing near the sideburn area above the zygomatic arch [24].

**Figure 9:**
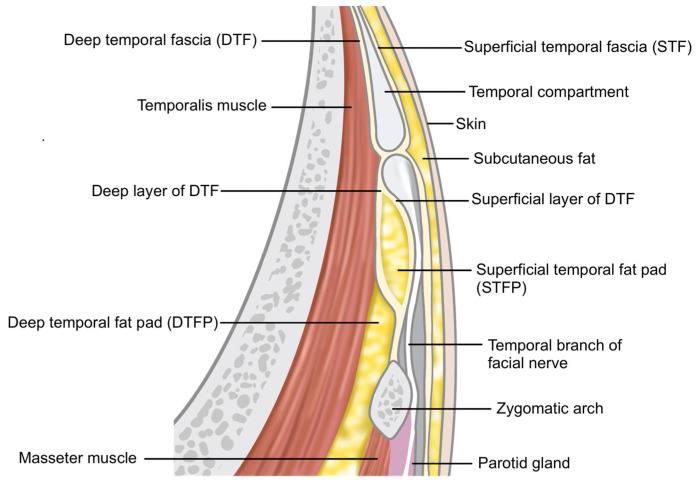
Position of the temporal branch of the facial nerve.

When considering the depth of traversal, the temporal branch of the facial nerve emerges from the parotid gland and initially travels beneath the deep fascia known as the parotid masseteric fascia [28,31,32]. As it crosses above the zygomatic arch, the nerve penetrates the deep fascia of the temple, the deep temporal fascia (DTF), and continues along the underside of the superficial temporal fascia (STF) before branching towards the frontalis muscle. In the superficial segments of its course, particularly, caution is necessary (Figure 9) [17]. Typically, the temporal branch runs just beneath the STF starting approximately 2–3 cm above the upper border of the zygomatic arch and about 1–1.5 cm lateral to the orbital rim. Between this point and the arch, it is known that the zygomaticotemporal nerve, a sensory nerve of the temple region, emerges through a foramen in the bone [28].

**Figure 10:**
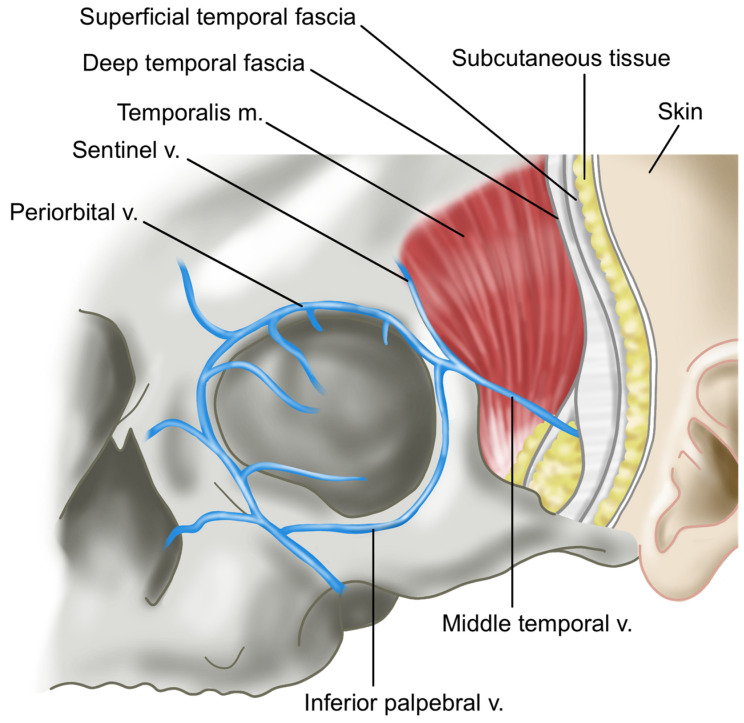
Location of the middle temporal vein.

The major veins in the temporal region, the sentinel vein, and the middle temporal vein are among the largest veins in the face, and improper injection of fillers into these vessels can potentially result in serious complications such as cavernous sinus thrombosis or pulmonary embolism [11]. The middle temporal vein is located within the superficial temporal fat pad (STFP), which lies between the superficial and deep layers of the DTF. This large vein, with a diameter of approximately 5–10 mm, runs horizontally about 2 cm above the upper border of the zygomatic arch and drains into the superficial temporal vein. If filler inadvertently enters this vein, there is a risk of pulmonary embolism via the external jugular vein [22]. There have been reports of fat embolisms occurring in cases of fat grafting when fat entered this vessel (Figure 10) [33].

The sentinel vein, also known as the medial zygomaticotemporal vein, is a branch of the internal maxillary vein with a diameter of approximately 2–3 mm. This vein vertically penetrates the temporalis muscle, deep temporal fascia (DTF), and superficial temporal fascia (STF). Typically, the point where it enters vertically is located about 5 mm lateral to the zygomaticofrontal suture line and approximately 2.5 cm from the lateral canthus. During facelift surgery, when dissecting between the STF and DTF, this vein often appears as a purple column, visible between the two fascia layers [14].

**Figure 11:**
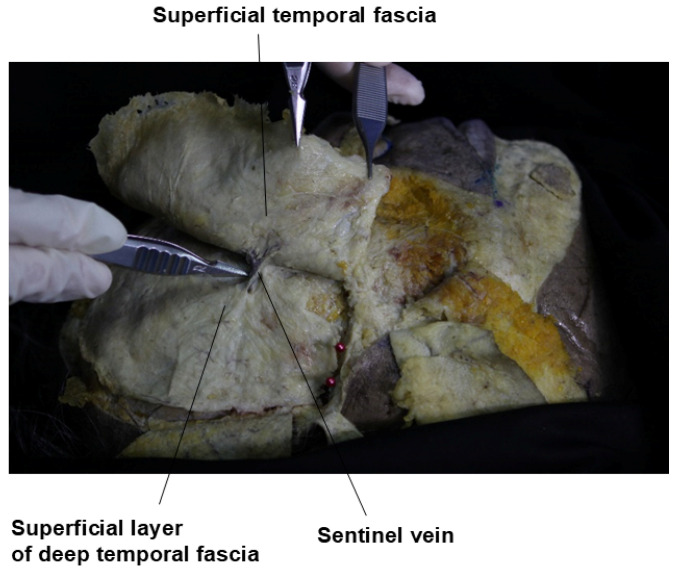
Location of the sentinel vein.

After passing through the DTF and emerging at the STF, the sentinel vein branches and connects with other veins traveling towards the outer eyebrow and temporal crest, following the subcutaneous fat layer [34]. In individuals with thin skin, this vein can become more prominent when the head is lowered or during the Valsalva maneuver, which increases venous pressure in the head, allowing the vein’s course to be more easily identified. Occasionally, when a significant volume is added to the temporal region during filler procedures, the subcutaneous branches of the sentinel vein may become more pronounced post-procedure (Figure 11) [35].

**Figure 12:**
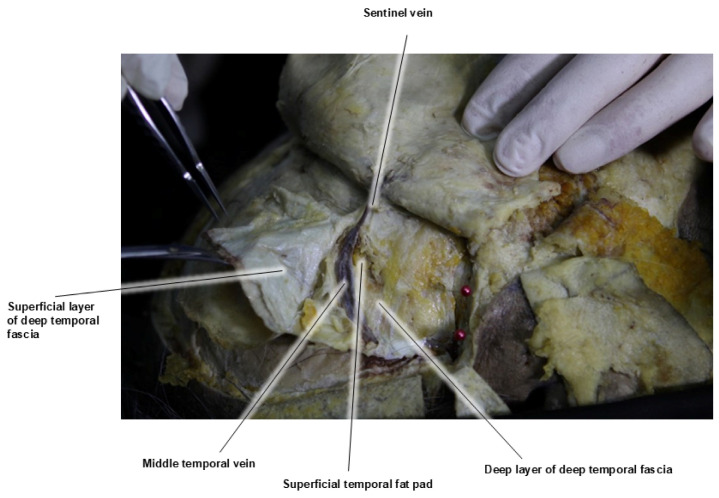
Relationship between the sentinel vein and the middle temporal vein.

**Figure 13:**
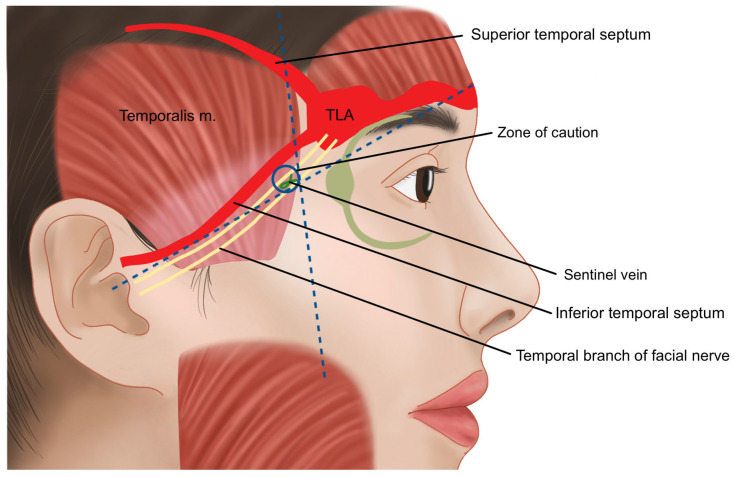
Zone of caution for the sentinel vein and the temporal branch of the facial nerve.

In the past, identifying the vertical column of the sentinel vein during facelift surgery was considered a reliable method for locating the underlying temporal branch of the facial nerve, serving as a landmark for facial nerve identification. However, recent insights suggest that the facial nerve often runs parallel to the inferior temporal septum (ITS) and is more commonly found above the sentinel vein, challenging its effectiveness as a nerve indicator. Clinically, the sentinel vein converges with the middle temporal vein to form the periorbital vein, which then connects with the supratrochlear and supraorbital veins [36]. These veins eventually lead to the superior ophthalmic vein, which is linked to the cavernous sinus, a cavity at the base of the skull. This anatomical pathway implies that improper injection of fillers or fat into this vein could result in serious complications such as cavernous sinus thrombosis (Figure 12) [37]. Additionally, the superficial distribution of veins in the subcutaneous tissue near the point where the sentinel vein penetrates the fascia is a critical area where branches of the facial nerve also ascend superficially. The proximity of these two structures creates a “caution zone,” particularly around a circular area with a diameter of approximately 1 cm. This zone is located at the intersection of a line drawn from the helical root of the ear to the inner margin of the superior orbit, and another line extending from the lateral orbital rim’s outer margin. When navigating with a cannula in this area, it is essential to proceed with utmost care to avoid damaging these closely situated structures (Figure 13) [38].

**Figure 14:**
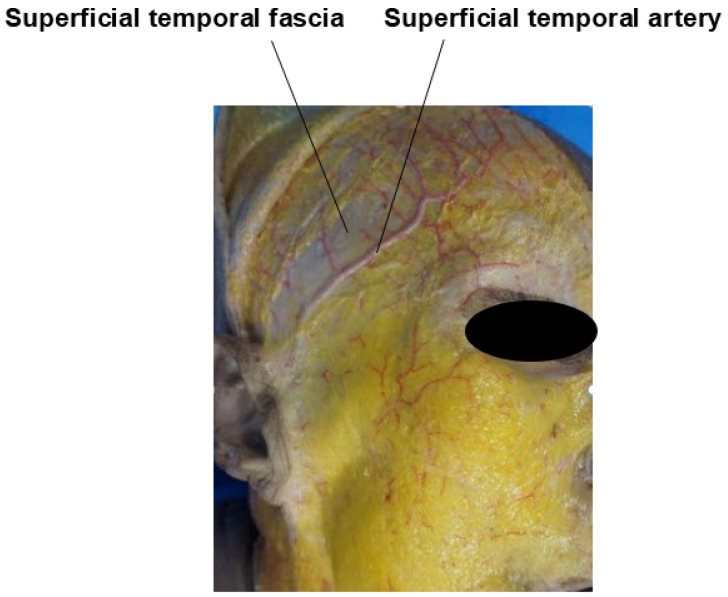
Location of the superficial temporal artery on the superficial temporal fascia.

When considering the depth at which anatomical structures like arteries and branches of the facial nerve traverse, it is generally understood that these structures course at the level of the superficial temporal fascia (STF). However, in cadaver dissections, one can observe that these arteries and nerves are often enveloped by the fascia, creating the appearance that they are running on top of the fascia while bulging into the subcutaneous fat layer. This observation has led to descriptions suggesting that the vessels and nerves travel superficially to the fascia (Figure 14) [10]. To minimize the risk of damaging these structures during cannula-based procedures, it is crucial to have a thorough understanding of the pathways these structures follow. When the cannula needs to cross these pathways, the non-dominant hand should be used to pinch the skin and underlying tissue, ensuring that the cannula enters at the correct plane. This technique is especially important in patients with well-developed ligaments where the adhesion between the skin and underlying tissues is pronounced, necessitating precise plane targeting during the procedure [35].

The sentinel vein and the zygomaticotemporal nerve, which is responsible for sensation, emerge deeply from the temporal bone and gradually become more superficial as they approach the orbit. As long as the points of origin from the bone are identified and approached with caution, these structures should not pose significant issues. Similarly, the middle temporal vein, which lies between the superficial and deep layers of the deep temporal fascia (DTF), can be navigated safely if the cannula moves gently through the areolar tissue beneath the STF, minimizing the risk of injury [11].

## 4. Procedure Method—Injection Plane

**Figure 15:**
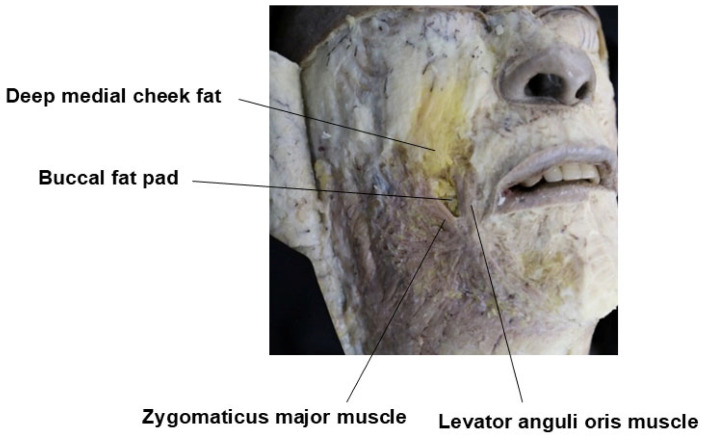
Buccal fat pad is an extension of the deep temporal fat pad.

With age, weight loss, or naturally, the volume of the temporalis muscle and the superficial temporal fat pad (STFP) decreases. Additionally, as aging progresses, the volume loss of the buccal fat pad, which extends from the deep temporal fat pad (DTFP) and supports the temporal region from below, further exacerbates the volume reduction in the temporal area. This, combined with the decreased elasticity of the skin and soft tissues, causes the cheeks and perioral areas to sag, making volume restoration in the temporal region even more necessary (Figure 15) [21].

When considering the spaces available for filler injection, the subcutaneous layer is generally not ideal for the temporal region, as it lacks significant subcutaneous fat, making it difficult to achieve a smooth and even filler distribution, thus leading to a higher likelihood of post-procedure irregularities. The optimal spaces for volumizing injections are between the fascial layers and beneath the muscle. Typically, when the volume deficit is not severe, the compartment between the superficial temporal fascia (STF) and the deep temporal fascia (DTF) is suitable [35]. For more substantial volume restoration, injecting into the STFP between the superficial and deep layers of the DTF or beneath the temporalis muscle is recommended. However, injecting into the space between the DTF and temporalis muscle (DTFP) can be challenging due to the difficulty in accurately targeting this layer. Intramuscular injections are also not ideal, as they pose a risk of injuring branches of the deep temporal artery and vein and may result in a shorter duration of effect due to muscle movement, making it a less desirable injection plane (Table 2) [22].

## 5. Procedure Method—Injection Point

**Figure 16:**
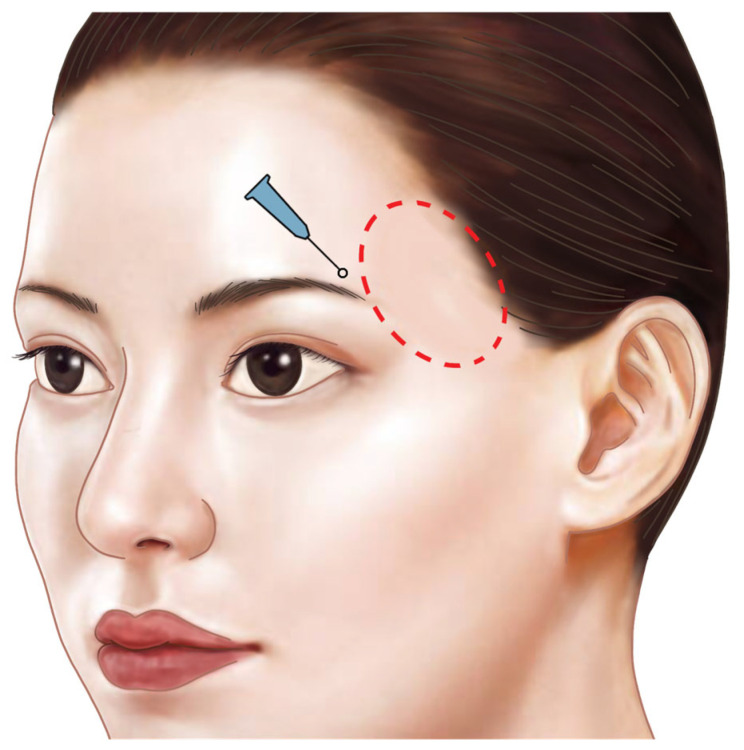
Injection entry point and technique for the cannula: the first injection entry point for the anterior main depression is located at the upper margin of the eyebrow on the lateral orbital rim line around the border of the forehead and temple. The injection technique involves the retrograde horizontal fanning technique.

The procedural guideline is an expert opinion with a review of anatomical literature and clinical techniques rather than presenting new empirical research. In cases of temporal hollowing, it is typically the anterior two-thirds of the temporal region that experiences volume loss. Therefore, an injection point should be created to restore volume in this area using a cannula. For mild to moderate volume restoration, a puncture site can be made approximately 1 cm above the upper border of the brow at the boundary between the forehead and the temporal region (usually aligned vertically with the lateral orbital rim). This location avoids the main pathways of the anterior branch of the superficial temporal artery, the zygomatico-orbital artery, and the temporal branch of the facial nerve. Care should be taken to avoid any visible small venous branches under the skin during puncture (Figure 16) [39].

**Figure 17:**
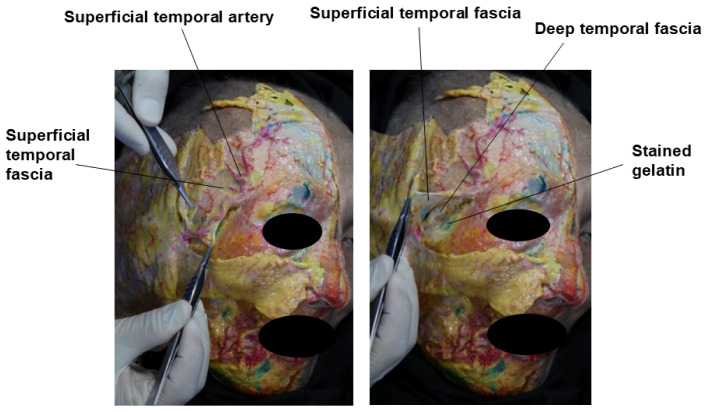
Space between the superficial and deep temporal fascia.

After inserting the cannula and encountering the first resistance at the superficial temporal fascia (STF), proceed carefully until reaching the deeper resistance at the deep temporal fascia (DTF). At this point, do not advance further; instead, gently guide the cannula along the space between the two fasciae, focusing on restoring the volume in the hollowed area by working within the upper and lower temporal compartments. During cannula advancement, it is important to remain parallel to the location of the inferior temporal septum (ITS) and avoid descending below it. This technique minimizes the risk of damaging critical nerves and vessels located beneath the ITS (Figure 17) [22].

In cases of severe temporal hollowing, the area just above the zygomatic arch often exhibits significant volume loss, necessitating substantial restoration. While the space between the temporalis muscle and the temporal bone has historically been the most commonly treated deep layer, it is now considered less ideal. The author’s preference is to inject filler into the superficial temporal fat pad (STFP) located between the superficial and deep layers of the deep temporal fascia (DTF) [21,24].

**Figure 18:**
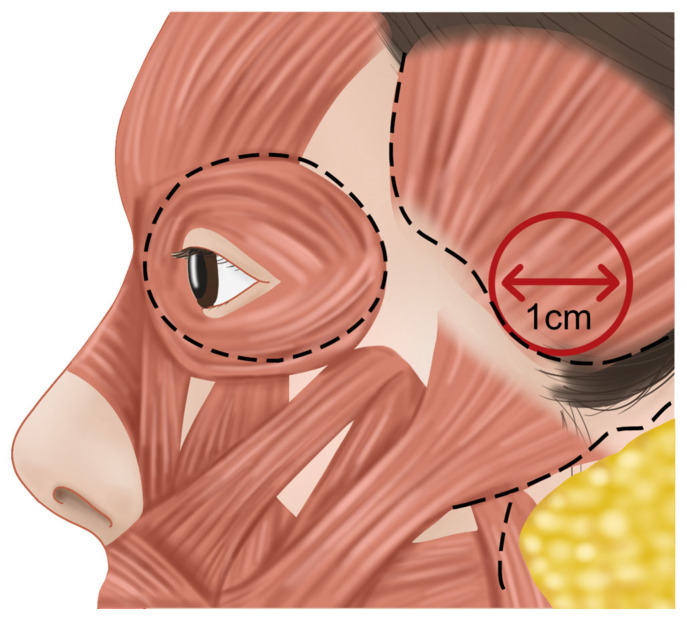
Safe boundary of the injection entry point for temporal hollowing, marked by a circle with a 1 cm diameter along the borderline of the upper margin of the zygomatic arch and the lateral margin of the lateral orbital rim.

For the injection point above the zygomatic arch, a virtual circle with a diameter of approximately 1 cm should be drawn at the intersection where the upper margin of the zygomatic arch meets the lateral margin of the lateral orbital rim. Within this circle, an injection point can be established that allows deep filler placement without risking damage to critical structures such as the superficial and deep temporal arteries, the facial nerve branches, the zygomaticotemporal nerve, the sentinel vein, and the middle temporal vein (Figure 18) [24].

**Figure 19:**
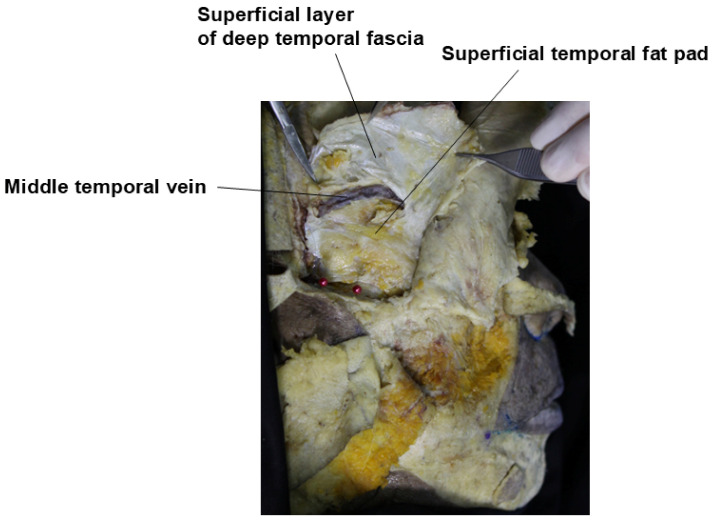
Location of the superficial temporal fat pad.

When injecting filler into the STFP, insert the cannula after the needle puncture, which will easily penetrate the first fascia, the STF. The next resistance encountered is the superficial layer of the DTF. Applying a moderate amount of force to pass through this fascia, the cannula will reach the deep layer of the DTF, which is notably firm. Carefully advance the cannula along this very firm deep layer, allowing for the injection of filler within the STFP located between the superficial and deep layers. To minimize contact with the middle temporal vein, keep the cannula close to the floor and focus on restoring volume within the 2 cm range above the zygomatic arch (Figure 19). For areas above this range, where the hollowing is typically less severe, switch the injection plane to the compartment between the STF and DTF [17].

**Figure 20:**
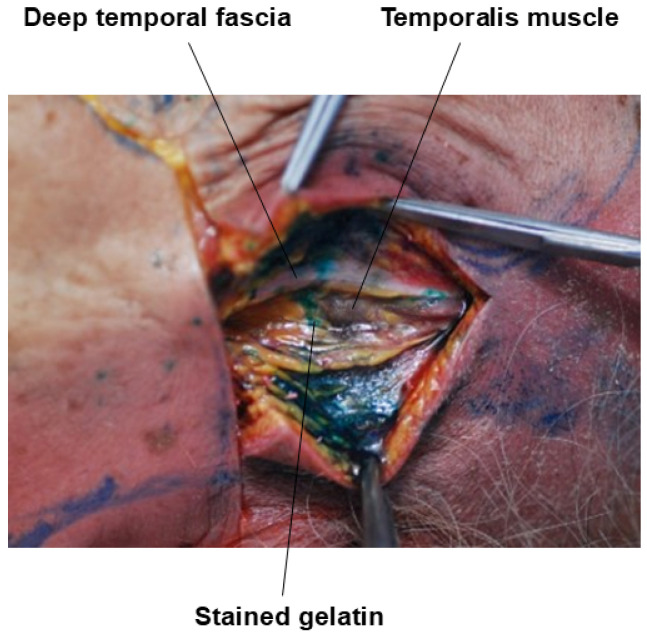
Gelatin injection under the temporalis muscle.

When attempting to penetrate the deep layer of the deep temporal fascia (DTF) and inject filler beneath the muscle, the use of a needle is generally more effective than a cannula due to the need to pierce through a very firm layer. Within the previously mentioned safe zone, the needle should be inserted vertically to penetrate the dense fascial layer and then advanced through the muscle until it contacts the bone [39]. At this point, filler can be injected along the temporal bone beneath the muscle. Given that this area often requires a substantial amount of high-viscosity filler to restore volume effectively, it is important to consider the type and quantity of filler to be used prior to the procedure (Figure 20) [40].

**Figure 21:**
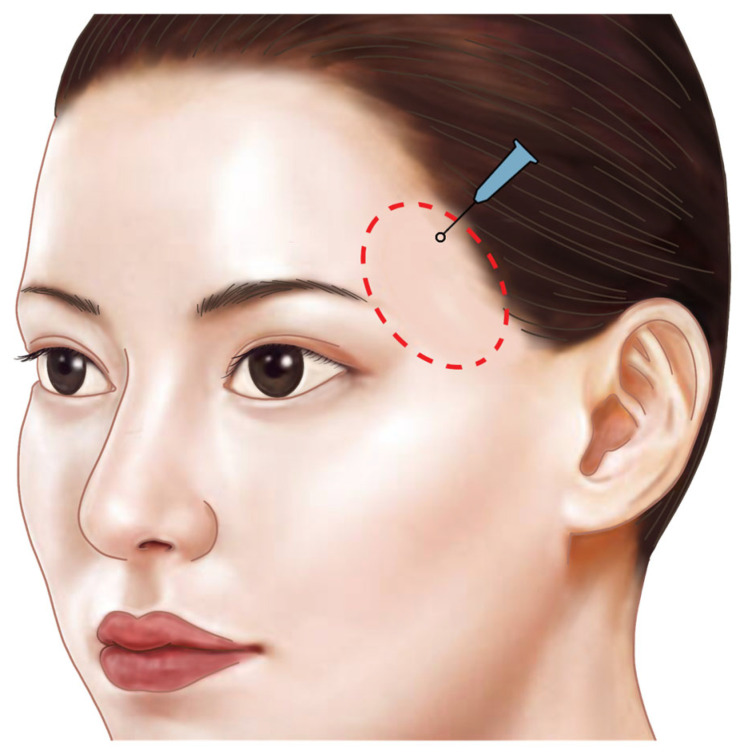
Injection entry point and technique for posterior depression: the second injection entry point, if necessary, is at the anterior hairline point (about 1 cm above the eyebrow level). The injection technique involves the retrograde horizontal fanning technique.

When addressing the main depression in the anterior two-thirds of the temporal region while also needing to correct the posterior one-third, it is advisable to create a new entry point at the posterior site rather than extending from the anterior entry point. Since posterior hollowing typically requires less volume, it can be adequately addressed by restoring the upper temporal compartment located between the superficial temporal fascia (STF) and DTF [11,24]. To avoid damaging nerves or blood vessels, an entry point can be established approximately 1 cm above the hairline at the intersection where a horizontal line drawn from the upper margin of the eyebrow meets the hairline (Figure 21). Similar to the anterior volume restoration, after performing a needle puncture, a cannula is inserted to position the filler within the compartment between the STF and DTF, focusing on restoring the posterior volume [39].

**Figure 22:**
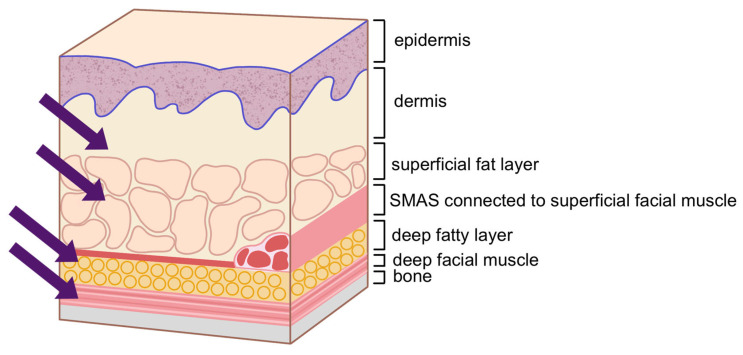
Injection planes, products, and volume. Injection planes include subfascial injection between the superficial temporal fascia (TPF) and deep temporal fascia (DTF) using a cannula; injection into the superficial temporal fat pad (STFP) between the superficial and deep layers of DTF using a cannula; and submuscular injection by needle when the fat pad layer is scarce. Products and volume used include Restylane Volyme or Lyft (1–1.5 mL for each area) with a 23G needle or cannula, and if necessary, 0.5–1 mL of Restylane Refyne or Vital for subdermal injection to even out the surface and the shoulder margin of the augmented area.

Following the volume restoration, if there are visible surface irregularities or noticeable boundaries around the enhanced areas, these can be smoothed out by injecting a softer filler into the subdermal layer, including the dermis, to achieve a more even surface and complete the procedure (Figure 22) [11].

When performing filler injections beneath the temporalis muscle using a needle, some practitioners advocate for a straightforward approach by selecting the deepest point of the temporal fossa as the entry point and avoiding intravascular injection by locating the pulse of the superficial temporal artery and inserting the needle deeply away from the palpable pulse. While it is true that the main branch of the superficial temporal artery is easily palpable in front of the ear, the anterior branch, which runs towards the eyebrow, can be difficult to detect in the temple area [17]. Moreover, this technique poses risks not only to the anterior branch of the superficial temporal artery but also to the temporal branch of the facial nerve, the middle temporal vein, and the deep temporal artery and vein within the temporalis muscle. As such, this method cannot be considered a safe approach [10].

Furthermore, it is often suggested that after the needle has passed through the muscle and reached the temporal bone, filler can be safely injected by feeling for the periosteum covering the bone. However, cadaver studies reveal that, unlike other areas, the space between the temporalis muscle and the temporal bone lacks a thick periosteum, with the muscle attaching directly to the bone. Therefore, when attempting to move the needle based on the assumption that it is gliding along the periosteum, there is a significant risk that the needle tip may actually move within the muscle, potentially damaging the intramuscular blood vessels [11]. Moreover, if the filler is deposited within the muscle, the strong contractions of the temporalis muscle, which is primarily responsible for mastication, may cause the filler to shift, diminishing its effectiveness. Additionally, recent concerns have highlighted that repeated muscle contractions may alter the rheological properties of the filler, particularly its elasticity, which is essential for maintaining the structure and support in the treated area. Consequently, injecting filler between the temporalis muscle and the temporal bone is no longer widely recommended as a preferred technique [10].

## 6. Discussion

The anatomy-based approach to temporal filler injections highlights the importance of selecting the correct injection plane to achieve optimal aesthetic outcomes. The choice of plane—subfascial, within the superficial temporal fat pad, or submuscular—depends on the patient’s anatomy and the desired effect. Subfascial injections, placed between the superficial temporal fascia (TPF) and deep temporal fascia (DTF), provide a stable and safe zone for volumization with minimal risk of vascular injury. This approach helps avoid visible lumps and irregularities, particularly in patients with thin or atrophic temporal fat pads [41,42,43,44].

Injecting within the superficial temporal fat pad offers a natural, soft contour by filling the space between the superficial and deep layers of the DTF. This technique is especially useful when subtle volume enhancement is needed without disrupting deeper anatomical structures. However, care must be taken to avoid the sentinel vein and temporal nerve branches, which are prone to injury if injections are placed incorrectly. Additionally, submuscular injections are employed when the temporal fat pad is insufficient, requiring direct volumization beneath the temporalis muscle. These injections offer deeper augmentation but necessitate precise techniques to prevent complications such as vascular compression or nerve irritation [45,46,47,48,49,50,51,52].

Patient-specific anatomical variations, including the location of the superficial temporal artery, middle temporal vein, and zygomatico-orbital artery, must be carefully assessed to guide safe injection practices. Using a cannula instead of a needle minimizes the risk of vascular injury, while retrograde fanning techniques ensure even distribution of the filler. Combining volumizing fillers like Restylane Volyme or Lyft with softer fillers such as Restylane Refyne for superficial contouring helps achieve a smooth and natural transition across treated areas. This multifaceted approach underscores the importance of detailed anatomical knowledge and tailored injection strategies to enhance temporal aesthetics while minimizing the potential for adverse events.

This study’s primary strength lies in its integration of anatomical knowledge with clinical techniques, providing a practical, anatomy-based framework that is both comprehensive and accessible for practitioners. By combining expert experience with a review of current anatomical literature, the guide offers clear, step-by-step guidance on filler injection techniques that prioritize safety and efficacy in the anatomically complex temporal region. This approach is particularly valuable for clinicians seeking to minimize complications related to vascular and nerve structures.

However, the study is limited by its nature as an expert opinion and clinical guide rather than a systematic review or empirical study. This means that while the recommendations are based on clinical experience and existing anatomical insights, they may not reflect new, controlled, experimental data or outcomes from broad patient populations. Future empirical studies or clinical trials could further validate these techniques, providing quantitative evidence of their efficacy and safety across diverse patient groups.

## 7. Conclusions

In conclusion, this expert guide offers clinicians a detailed, anatomy-based approach to filler injections in the temple region, emphasizing both aesthetic enhancement and patient safety. By understanding the complex anatomical structures—particularly the vascular and neural landmarks in the temporal area—practitioners can select appropriate injection planes and techniques that minimize risks. The use of planes such as subfascial, within the superficial temporal fat pad, and submuscular allows tailored treatment based on individual anatomical variations. Through a methodical approach that integrates anatomical insights with clinical expertise, this guide aims to improve treatment outcomes, helping clinicians achieve natural, rejuvenated facial contours while avoiding common complications associated with temporal filler injections.

## Figures and Tables

**Table 1:** Temporal layer compared to other facial regions.

Basic layer	Midface	Temple
Skin	Skin	Skin
Subcutaneous tissue	Superficial fat compartment	Lateral temporal cheek fat
Musculo-aponeurotic layer	SMAS	Superficial temporal fascia (STF) or temporoparietal fascia (TPF)
Loose areolar tissue (LAT)	Deep fat compartmnet	Upper temporal compartment (UTC) or innominate fascia & Lower temporal compartment (LTC) or fibrofatty extension, parotid temporal fascia (PTF)
Periosteum	Facial muscles	Superficial & deep layer of deep temporal fascia (DTF) or temporalis fascia (TF)

**Table 2:** Possible injection planes.

1. Subdermal and superficial fat layer
2. Upper and lower temporal compartments or innominate fascia between the superficial and deep temporal fascia
3. STFP(superficial temporal fat pad) between superficial and deep layer of deep temporal fascia
4. DTFP (deep temporal fat pad) between deep temporal fascia and temporalis muscle
5. Temporalis muscle
6. Space between temporalis muscle and temporal bone
